# New Allogeneic Hematopoietic Stem Cell Transplantation Method: Hematopoietic Stem Cell Transplantation Plus Thymus Transplantation for Intractable Diseases

**DOI:** 10.1155/2013/545621

**Published:** 2013-05-12

**Authors:** Naoki Hosaka

**Affiliations:** ^1^Department of Pathology, Kansai Medical University Kori Hospital, 8-45 Korihondori, Neyagawa, Osaka 572-8851, Japan; ^2^Department of Clinical Sciences and Laboratory Medicine, Kansai Medical University, 2-5-1 Shinmachi, Hirakata City, Osaka 573-1010, Japan

## Abstract

Although allogeneic hematopoietic stem cell transplantation (allo-HSCT) has become a valuable strategy for some intractable diseases, a number of problems remain to be resolved. We have developed a new HSCT method, HSCT + thymus transplantation (TT) from the same donor, which induces elevated T cell function with mild graft-*versus*-host disease (GVHD) in comparison to conventional HSCT alone and HSCT + donor lymphocyte infusion (HSCT + DLI). This new method is effective in the treatment of several intractable diseases and conditions, such as autoimmune diseases in aging, advanced malignant tumors, exposure to supralethal irradiation, multiple organ transplantation from different donors, and type 2 diabetes mellitus, for which conventional methods are ineffective. Our findings suggest that allo-HSCT + TT is preferable to conventional allo-HSCT alone or allo-HSCT + DLI. This method may become a valuable next-generation HSCT technique.

## 1. Introduction

In recent years, allogeneic hematopoietic stem cell transplantation (allo-HSCT) has become a valuable strategy for the treatment of hematological disorders (leukemia, lymphoma, and aplastic anemia), congenital immunodeficiencies, autoimmune diseases, metabolic diseases, and malignant tumors [1–7]. However, there are still a number of problems associated with these methods. Although T cells in the graft facilitate engraftment, they often induce graft-*versus*-host disease (GVHD) [8]. Conversely, if the antihost reaction is low, hematopoietic failure and/or primary disease recurrence may occur. In addition, the success rate of hematopoietic stem cell transplantation (HSCT) is low in elderly patients with low facility for engraftment and/or risk of severe complications [9–11]. Recent experience with radiation accidents also indicated that HSCT alone is ineffective for patients exposed to supralethal doses of irradiation with severe organ damage [12, 13]. Therefore, it is extremely important to overcome these problems of allo-HSCT.

The thymus is the organ in which T cells are differentiated and produced with induction of tolerance to the host. The thymus also regulates biological homeostasis using the cells with several cytokines and hormones [14]. Therefore, allo-HSCT with cotransplantation of the thymus from the same donor may be a beneficial method, as another site of allo-T cell production. Thymus transplantation (TT) itself has been used to treat immunodeficiency diseases, such as DiGeorge syndrome and HIV infection, and to increase T cell function [15, 16]. TT is also effective in age-related diseases with correction of immune dysfunctions in mice [17]. To our knowledge, however, there have been no previous studies regarding the efficacy of HSCT + TT in treatment of intractable diseases.

We recently developed a method of allo-HSCT in conjunction with TT from the same donor [18–27]. This method results in elevated T cell function with mild GVHD compared to HSCT alone or HSCT + donor lymphocyte infusion (HSCT + DLI) [18]. The mechanism underlying these effects involves not only CD4^+^ FoxP3^−^ effector T cells (T_eff_  cells) but also CD4^+^ FoxP3^+^ regulatory T cells (T_reg_  cells), which prevent GVHD and autoimmunity [28, 29], produced by the allotransplanted thymus. The percentages of these cells are intermediate between HSCT alone and HSCT + DLI. The percentage of T_reg_ cells in HSCT + TT is lower than that in HSCT alone, but higher than that in HSCT + DLI, while the opposite is true for the percentage of T_eff_ cells [18]. We have examined application of this method for several intractable diseases. Our findings indicated that allo-HSCT + TT is preferable to the conventional allo-HSCT alone or allo-HSCT + DLI for several intractable diseases and conditions.

## 2. Results

### 2.1. Theory of Allo-HSCT + TT

**Figure 1:**
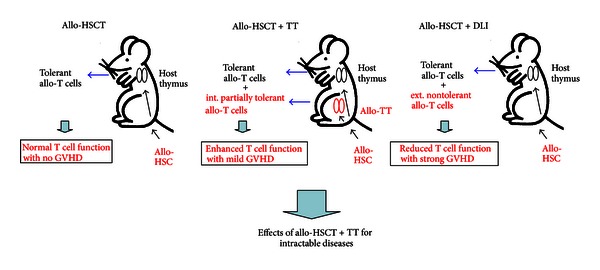
Theory of allo-HSCT + TT. In the case of conventional allo-HSCT (left), the allo-T cells develop and are tolerated in the host thymus, and no GVHD occurs. In the case of allo-HSCT + DLI (right), nontolerated allo-T cells are externally supplied, and strong GVHD is induced with reduction of T cell function. In the case of allo-HSCT + TT, the allo-T cells develop internally in the allothymus. The T cells show partial tolerance to the host, and only mild GVHD occurs with elevation of T cell function (middle).

First, we present the theory of allo-HSCT + TT (Figure 1). In the case of conventional allo-HSCT, allo-HSC is transferred into the host, and allo-T cells develop in the host thymus. The T cells show induced tolerance toward the host with thymic antigen-presenting cells and/or epithelial cells [30] and do not induce GVHD with normal T cell function (Figure 1, left). In contrast, nontolerant allo-T cells are externally supplied in the case of HSCT + DLI, resulting in strong GVHD, and the T cell number and function finally decrease (Figure 1, right). In the case of HSCT + TT, allo-T cells develop internally from the transplanted allothymus in the host. Interestingly, the T cells are partially tolerant to the host and induce low GVHD. In addition, the T cell function increases.

### 2.2. Application of Allo-HSCT + TT

#### 2.2.1. Autoimmune Diseases

We examined the effects of HSCT + TT in treatment of several intractable diseases (Table 1). Female MRL/lpr mice develop systemic lupus erythematosus- (SLE-) like lupus glomerular nephritis [31]. Bone marrow transplantation (BMT) alone is ineffective in these mice because of the radioresistance with Fas deficiency [32–34]. However, BMT + adult thymus transplantation (ATT) overcame these problems and induced donor-derived chimerism [19]. As a result, it also led to successful treatment of nephritis with reduction of serum autoantibodies and deposition of IgG in glomeruli. Aged female MRL/+ mice developed chronic pancreatitis with sialoadenitis [35]. HSCT (BMT or fetal liver cell transplantation (FLT)) is ineffective because of insufficient engraftment of donor cells with the involved host thymus (described below). However, HSCT (BMT or FLT) + fetal thymus transplantation (FTT) showed complete engraftment of donor cells and was effective in treating diseases with reduction of serum amylase [20]. These results suggest that these autoimmune diseases can be treated by replacement of the pathological hematopoietic system with HSCT + TT [36, 37]. 

#### 2.2.2. Malignant Tumors

We next investigated the effects of HSCT + TT in tumor-bearing mice [18]. Mice treated with BMT alone showed significant tumor regression compared with untreated controls. Although mice treated with BMT + DLI showed greater tumor regression than those treated with BMT alone, strong GVHD also occurred and they died at an early stage. Interestingly, mice treated with BMT + ATT showed less GVHD than those treated with BMT + DLI, even with a comparable level of tumor regression. 

We also examined the effects of HSCT + TT on leukemia [22]. In contrast to solid tumors, most mice treated with BMT + ATT or BMT + DLI showed almost complete remission of the tumor with long-term survival compared to those treated with BMT alone. However, the level of GVHD in those treated with BMT + ATT was significantly lower than that in those treated with BMT + DLI. These results suggest that BMT + ATT may be effective in treatment of not only solid tumors but also leukemia, without increased risk of GVHD.

The thymus atrophies with bone marrow suppression in hosts with advanced tumors, thus causing immunodeficiency, which is one of the major causes of death in such cases [38, 39]. We further examined the effects of HSCT + TT in mice bearing advanced tumors [21]. Although the thymus still atrophied in mice treated with allo-BMT + FTT, the transplanted fetal thymus had grown well. These mice showed longer-term survival than those treated with syngeneic- (syn-) or allo-BMT alone, or syn-BMT + FTT with inhibition of lung metastasis. Interestingly, third-party FTT was also effective (described below as triple chimera). These findings suggest that HSCT + TT may also be effective for long-term survival in advanced tumors.

#### 2.2.3. Aging

Advanced age is one of the risk factors for unsuccessful BMT [9, 10]. The major reason for this is the thymic involution leading to insufficient T cell production and function, as described above. We examined the effects of BMT + FTT in aged models: senescence-accelerated mouse P1 strain (SAMP1) [40] and aged MRL/+ mice (same model as no. 1) [20, 23]. Both strains of mice treated with HSCT + TT showed significantly longer survival than those treated with HSCT alone. Although donor cells could not be engrafted into the host by BMT alone, resulting in hematopoietic failure and early death, the cells could be engrafted sufficiently by BMT + FTT. Interestingly, these hosts with long-term survival also showed elevated T cell function compared to either untreated mice or those treated with HSCT alone, and the level was comparable to that in normal mice. These results suggest that BMT + FTT is effective not only for engraftment of donor cells but also in restoring immune function in aged hosts. 

#### 2.2.4. Use of Third-Party Thymus Tissue

**Figure 2:**
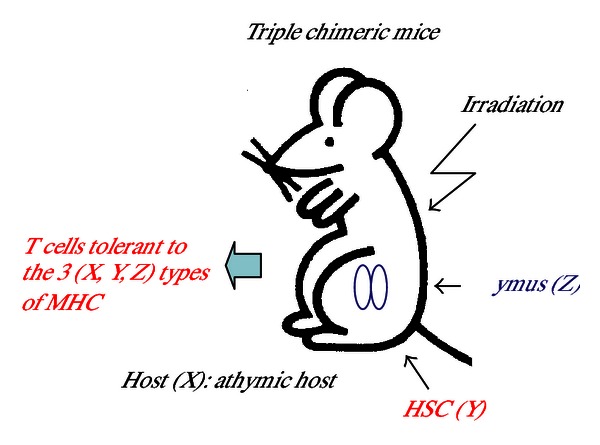
Use of third-party thymus tissue in triple chimera. Lethally irradiated athymic nu/nu mice (host: X) were transplanted with allogenic HSC (donor: Y) and third-party thymus (donor: Z) as triple chimeras.

Transplantation of multiple organs from different donors is desirable in patients with several intractable diseases. Although grafting of the thymus itself can also induce tolerance [41], its effects on further tolerance at allo-HSCT have not been examined. Therefore, we examined triple chimeras consisting of lethally irradiated athymic (nu/nu) (X) mice transplanted with allo-BMC (Y) and third-party fetal thymus (Z) with a different major histocompatibility complex (MHC) type from both BMC and host type (Figure 2). The mice showed tolerance to all three MHC types—host, BMC, and the grafted thymus—but not toa fourth foreign MHC type [23]. We further examined the triple chimera with the above aged mouse model, SAMP1 mice, and the same results of tolerance for three MHC types were obtained. Although this model is limited to hosts with low thymic function, these results suggest that HSCT + TT can also be applied for multiple organ transplantation from different donors using third-party TT. 

#### 2.2.5. HSCT Conditioning

Reduction of the intensity of the HSCT conditioning regimen will decrease the side effects for both host and donor. Therefore, we examined the effects of HSCT and TT for two conditions, that is, low-dose irradiation (sublethal irradiation (SubLI)), which attenuates host damage, and low numbers of BMC (injection of low numbers of BMC (ILNBMC)), which reduces the burden on the donor [24]. BMT + DLI showed the shortest survival due to severe GVHD under both conditions, and BMT alone also showed short survival due to hematological failure with insufficient engraftment in ILNBMC. However, BMT + ATT was preferable for long-term survival with reduction of GVHD to BMT + DLI or with donor-derived chimerism to BMT alone. Next, we investigated the effects of HSCT and TT on supralethal radiation exposure [25]. HSCT alone, such as BMT, newborn liver cell transplantation (NLT), or FLT, was almost completely ineffective in mice exposed to supralethal irradiation with severe intestinal injury and weight loss, as reported in humans [12, 13]. However, NLT + newborn thymus transplantation (NTT) rescued the animals with the greatest efficacy showing improvement of the injury and prevention of weight loss among the three types of HSCT + TT (BMT + ATT, NLT + NTT, and FLT + FTT).

#### 2.2.6. Type 2 Diabetes Mellitus

There is increasing evidence that both autoimmune and autoinflammatory mechanisms are involved in the development of not only type 1 diabetes mellitus (T1 DM) but also type 2 DM (T2 DM) [42, 43]. Therefore, we recently examined the effects of HSCT + TT in leptin receptor-deficient (db/db) mice, an animal model of T2 DM [44]. BMT + NTT could be used to treat diabetes with complete replacement of HSC showing normalized immune functions, although BMT alone showed insufficient treatment with incomplete replacement of HSC and dysregulated immune function [26]. These results suggest that BMT + NTT may be effective for treatment of T2 DM and that correction of the pathogenic immunological function is important for treatment.

## 3. Discussion

As described above, allo-HSCT + TT is functionally superior to either conventional HSCT alone or HSCT + DLI for several intractable diseases and conditions. Elevation of T cell function with low GVHD facilitates engraftment of donor cells in HSCT + TT, and these allo-T cells may work more effectively and safely in treatment of several diseases compared to HSCT alone or HSCT + DLI.

**Figure 3:**
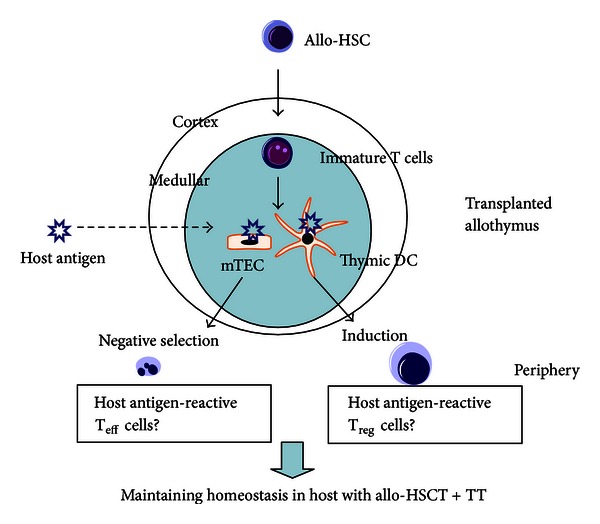
Immune regulation to maintain homeostasis by allo-HSCT + TT.

The elevation of T cell function in HSCT + TT is due to the newly developed T cells from the transplanted thymus [18]. In addition, the low GVHD is due to the partial deletion and induction of host antigen-reactive T_eff_ and T_reg_ cells from the transplanted allothymus. Although the detailed mechanism is not yet clear, it suggests that thymic antigen-presenting cells such as dendritic cells (DC) and/or medullary thymic epithelial cells (mTEC) in the transplanted thymus may play important roles with the antigen [30, 45–49] (Figure 3). As these T cells develop continuously in vivo, they do not induce lethal “autoimmune-like” GVHD to maintain homeostasis [50, 51]. Therefore, TT initially appears to represent a simple method but may represent a significant approach to supplying the organ in which T cells are differentiated, produced, and functionally regulated.

As the thymus shows functional differences with age, we also compared the effects of the thymus at three different ages (adult, newborn, and fetus) [25, 27]. Although HSCT + TT was superior to HSCT alone at all ages, NLT + NTT showed better results than FLC + FTT and BMT + ATT in these experiments. Although further studies are needed, these findings suggest that young thymus, as close as possible to newborn, may be preferable. 

However, it is both ethically and technically difficult to obtain adequate thymus tissue for clinical use. In this respect, grafts could be obtained from patients with congenital heart diseases or from aborted fetuses, as utilized previously [15, 16]. These materials are close to the preferred NT and may be used in third-party TT. In addition, a method for thymus regeneration and differentiation from stem cells has also been developed [52–57]. Therefore, HSCT + TT may become a viable strategy for the treatment of intractable diseases, conditions, or transplantation, and therefore this may become a valuable next-generation HSCT method.

## 4. Conclusion

The findings presented here indicate that allo-HSCT + TT is more effective against several intractable diseases compared with conventional allo-HSCT methods. This method may become a valuable strategy for the treatment of various diseases in humans.

## Figures and Tables

**Table 1:** Effective allo-HSCT + TT for various diseases and conditions compared with HSCT and HSCT + DLI.

Disease	Mouse model	TT	Results	Reference
(1) Autoimmune diseases				
(i) Lupus-like glomerulonephritis	MRL/lpr	ATT	Treatment of the glomerulonephritis	[19]
(ii) Chronic pancreatitis	MRL/+	FTT	Treatment of the chronic pancreatitis and sialoadenitis	[20]
(2) Malignant tumor				
(i) Early tumor	BALB/c with Meth-A sarcoma	ATT	Regression of tumor with low GVHD	[18]
(ii) Advanced tumor	BALB/c with Meth-A sarcoma	FTT	Long-term survival with inhibition of lung metastasis	[21]
(iii) Leukemia	B6 with EL-4 leukemia	ATT	Remission of tumor with low GVHD	[22]
(3) Aging	MRL/+	FTT	Long survival with elevation of T cell function	[20]
	SAMP 1	FTT	Same as above	[23]
(4) Use of third-party thymus tissue	B6, BALB/c, C3H (triple chimera)	FTT	Tolerance for 3 types of MHCs	[23]
(5) HSCT conditioning				
(i) Low numbers of BMC	BALB/c	ATT	Long survival with high donor chimerism and low GVHD	[24]
(ii) Low-dose irradiation	BALB/c	ATT	Same as above	[24]
(iii) Supralethal irradiation	BALB/c	NTT > FTT > ATT	Rescue with improvement of intestinal injury	[25]
(6) Type 2 diabetes mellitus	db/db	NTT	Treatment of the type 2 diabetes mellitus	[26]

HSCT: hematopoietic stem cell transplantation; TT: thymus transplantation; ATT: adult thymus transplantation; NTT: newborn thymus transplantation; FTT: fetal thymus transplantation.

## Abbreviations

Allo-HSCT: Allogeneic hematopoietic stem cell transplantation
ATT: Adult thymus transplantation
BMT: Bone marrow transplantation
DLI: Donor lymphocyte infusion
FLT: Fetal liver cell transplantation
FTT: Fetal thymus transplantation
GVHD: Graft-*versus*-host disease
HSCT: Hematopoietic stem cell transplantation
NLT: Newborn liver cell transplantation
NTT: Newborn thymus transplantation
T_eff_ cells: Effector T cells
T_reg_: Regulatory T cells
TT: Thymus transplantation
mTEC: Medullary thymic epithelial cells
DC: Dendritic cells.
